# Biosensors and Microfluidic Biosensors: From Fabrication to Application

**DOI:** 10.3390/bios12070543

**Published:** 2022-07-20

**Authors:** Madhusudan B. Kulkarni, Narasimha H. Ayachit, Tejraj M. Aminabhavi

**Affiliations:** 1 School of Electronics and Communication Engineering, KLE Technological University, Hubballi 580023, Karnataka, India; madhusudan.kulkarni@kletech.ac.in; 2 School of Advanced Sciences, KLE Technological University, Hubballi 580023, Karnataka, India; ayachit@kletech.ac.in

**Keywords:** biosensor, electrochemical, miniaturization, microfluidics, fabrication, nanomaterials, point of care (POC)

## Abstract

Biosensors are ubiquitous in a variety of disciplines, such as biochemical, electrochemical, agricultural, and biomedical areas. They can integrate various point-of-care applications, such as in the food, healthcare, environmental monitoring, water quality, forensics, drug development, and biological domains. Multiple strategies have been employed to develop and fabricate miniaturized biosensors, including design, optimization, characterization, and testing. In view of their interactions with high-affinity biomolecules, they find application in the sensitive detection of analytes, even in small sample volumes. Among the many developed techniques, microfluidics have been widely explored; these use fluid mechanics to operate miniaturized biosensors. The currently used commercial devices are bulky, slow in operation, expensive, and require human intervention; thus, it is difficult to automate, integrate, and miniaturize the existing conventional devices for multi-faceted applications. Microfluidic biosensors have the advantages of mobility, operational transparency, controllability, and stability with a small reaction volume for sensing. This review addresses biosensor technologies, including the design, classification, advances, and challenges in microfluidic-based biosensors. The value chain for developing miniaturized microfluidic-based biosensor devices is critically discussed, including fabrication and other associated protocols for application in various point-of-care testing applications.

## 1. Introduction

Biosensors are attractive devices used in academia, industries, and research laboratories [1,2,3]; they have the exceptional ability to recognize a biological event on a transducing device using a signal proportional to the analyte concentration to quantify a biological or biochemical response. In physiological fluids such as blood, urine, saliva, tears, and sweat, biosensors monitor diseases, water contamination, and disease-causing bacteria, and also act as biomarkers [4]. Biosensors have opened up new horizons in recent years, emphasizing biological domains to benefit mankind, healthcare, food, and environmental monitoring [5]. They can also help in high-throughput screening, homeland security, food safety, agriculture, medicine, environment, and pharmacology [6]. Advances in microfluidics have facilitated the design and development of miniaturized devices to control, coordinate, and modify micro-volumes of fluid via microchannels ranging from 10 to 100 microns [7]. Microfluidics is an area of microscale laminar fluid flow for better heat and mass transport which has created important platforms in biomedical diagnostics [8,9]. Due to a large surface-to-volume ratio, microfluidic-based devices can operate the fluid management in microchannels by significantly lowering the reaction volume, thereby demonstrating the essential movement of thermal mass and temperature gradients more efficiently and effectively. Furthermore, low-castability is a key point in the design and fabrication of various biosensors.

Point-of-care testing (POCT) involves on-site testing or testing near the location where care is to be given with the improvisation of healthcare devices. One of the most significant components of POCT is a microfluidic device with an integrated readout device [10]. POCT-based microdevices require very little human intervention, minimizing human errors and using a minimum amount of power consumption in an affordable manner. Most miniaturized and automated microfluidic POCT devices can be packed and integrated into a unified interface, enabling sensitive, minimally invasive, and non-invasive devices to detect several biomarkers associated with a biological sample such as saliva, urine, and blood in a continuous, rapid, low-cost, and reliable manner [11,12]. Device miniaturization has now become more common, particularly in electrochemical and biosensing analysis. In electrochemical research, reducing the electrode size can influence the detection limits of electrochemical techniques, along with the needed sensitivity, selectivity, repeatability, and accuracy, thereby providing a low-cost approach with high-throughput analysis [13,14].

The present review critically discusses the evolution, design, working principles, classifications, and recent advances in miniaturized microfluidic-based biosensors. Various types of biosensors that can be designed and fabricated using multiple techniques such as CO_2_ lasers, 3D printers, and UV direct lasers boost the importance of microfluidic-based biosensors, thus challenging the associated developments of miniaturized biosensors with the potential for automation, integration, and miniaturization. Further possibilities to improvise the design and future scope of the field associated with miniaturized microfluidic-based biosensors are discussed.

## 2. Evolution of Biosensors

**Figure 1:**
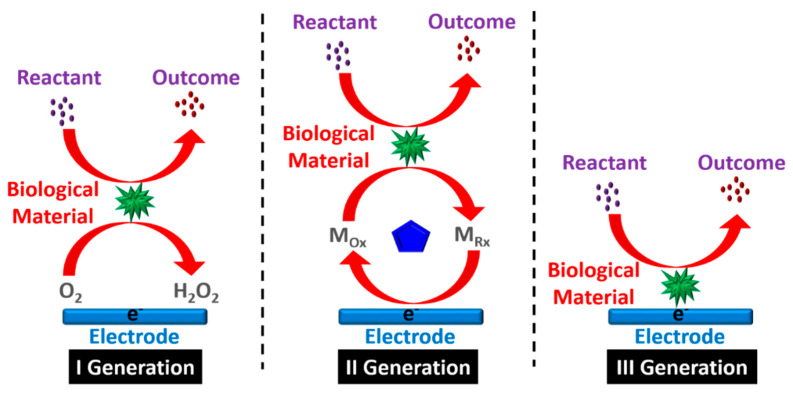
Three generations of the biosensor process.

Biosensors were first reported by Leland Charles Clark Jr. in 1962, who conceived the idea of demonstrating the components of a biosensor along with a strategy to integrate a bioreceptor with a transducer device [6]. Figure 1 shows three generations of the development of biosensors, while Table 1 summarizes the evolution pathway.

### 2.1. Design and Principles of Biosensors

**Figure 2:**
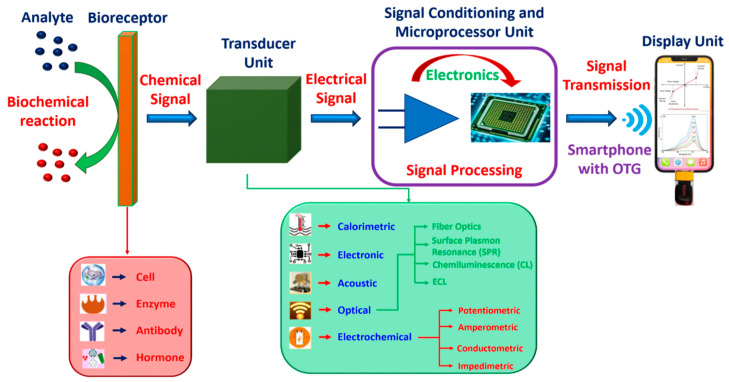
Schematics of a biosensor device consisting of various modules.

Biosensors can come in various sizes, shapes, and electrode materials, and can detect and evaluate viruses, pathogens, and diseases. They take the form of a small probe or an electronic device to generate an indicator that can be used to quantify further processing; the electronic device is responsible for communicating, recording, and detecting the changes in the physiological parameters of the biological or chemical components in the environment. The device includes (i) an analyte, (ii) biological material, (iii) a transducer, (iv) an electronic module, and (v) a display unit (see Figure 2). When the tested material is introduced into an electrolytic solution, its constituents are recognized (alcohol, glucose, lactose, and ammonia). Biorecognition creates a signal creation during the interaction between the analyte and the biological components, while the transducer transforms a biorecognition signal into a quantifiable electrical form, which indicates the presence of a biological or chemical objective. The analyte–bioreceptor interactions are proportional to the electrical or optical signals produced by the transducers, which can be connected to the cloud server to access and store the data; the output data can take the form of graphical, numerical or tabular analyses [6].

### 2.2. Classification of Biosensors

**Figure 3:**
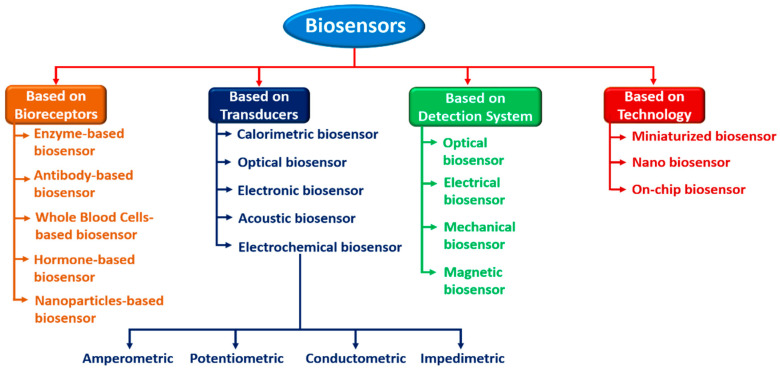
Classification of biosensors depending on the diverse use of bioreceptors and transducers.

Sensors can be divided into several categories based on their components or analytes to be detected. Active sensors must be powered from a separate source such as a thermistor, microphone, or strain gauge, which are also called parametric sensors. Passive sensors produce signals but do not need any external power source to operate; photodiode, piezoelectric, thermocouple, and thermistor sensors are some examples. Contact-based sensors such as temperature sensors and mechanical sensors necessitate physical contact with stimuli, whereas contactless sensors such as magnetic and optical sensors do not need any physical contact. Absolute sensors respond to stimuli on a vast scale, such as strain gauges and thermistors. The environmental stress determines the pressure, while the relative sensors detect the catalyst in relation to a fixed or changing reference, such as a thermocouple measuring the heat difference. An analog sensor is a device that converts the continuously changing physical parameter into an analog signal. Analog sensors include temperature, humidity, and pressure, to name a few. In a digital sensor, the output is in Boolean format. Depending on the type of signal detection, these sensors are classified as (i) chemical, (ii) thermal, (iii) physical, and (iv) biological. These have been widely employed in biomedical fields, as well as in the MEMS domain [35]. Figure 3 shows the classification of various types of biosensors depending on the diverse use of bioreceptors and transducers.

#### 2.2.1. Based on Bioreceptors

Biosensors are classified as catalytic or non-catalytic, based on the biorecognition concept. The interaction between analyte and bioreceptor in a catalytic biosensor results in a unique biochemical product reaction. Tissues, whole cells, bacteria, and enzymes are all included under this type of biosensor. The analyte is irreversibly coupled to the receptor in a non-catalytic biosensor, and no new biochemical reaction product is formed during the interaction. To identify the target, these sensors interact with nucleic acid, antibodies, and cell receptors.

##### Enzymes, Antibodies, Whole Cell, and Hormone-Based Biosensors

Enzymes are biocatalysts that can increase the rate of biochemical reactions in the bio-domain [36]. Other methods that are used in the recognition of the analyte include: (i) the enzyme metabolizing the analyte and obtaining the concentration by determining the catalytic conversion of the analyte; (ii) the analyte restraining or stimulating the enzyme where the concentration of the analyte is linked to reducing the formed enzymatic product; (iii) monitoring the changes in enzyme characteristics. Antibodies have been employed for several decades in a biomedical area [37], and these sensors can be implanted onto the antibodies or ligands that can affect antibody–antigen interactions. These are further divided into two categories: (i) non-labelled and (ii) labelled.

Enzyme-labeling is a method used in bioanalysis to place a chemical marker on a molecule within a substance. Enzyme-labeled antibodies are used for ELISA, Western blotting, and immunostaining. Labeled antibodies are detected by reaction with a substrate that emits light or changes color. Antibodies, like other proteins, can be labeled with small molecules, radioisotopes, enzymatic proteins, and fluorescent dyes.

Fungi, bacteria, viruses, and algae are used in whole cell-based biosensors because these biosensors have possible biological components in their structure. Without the need for extraction or purification, these microorganisms can self-replicate and produce recognition components such as antibodies. Cells can interrelate with a wide spectrum of analytes in this environment, resulting in an electrochemical reaction that a transducer can quickly detect and transmit.

Hormones are typically secreted by glands or certain types of cells that enter the bloodstream, and are specifically designed to target cells. Hormone electrochemical biosensing has become a tool for diagnosing and treating human diseases. The human body produces extremely few hormones that govern and control the metabolism, prompting efforts to create sensitive technology to identify them and overcome the shortcomings of other well-established methods (such as ELISA) in terms of selectivity, sensitivity, and time performance.

##### Nanoparticles (NPs)

**Figure 4:**
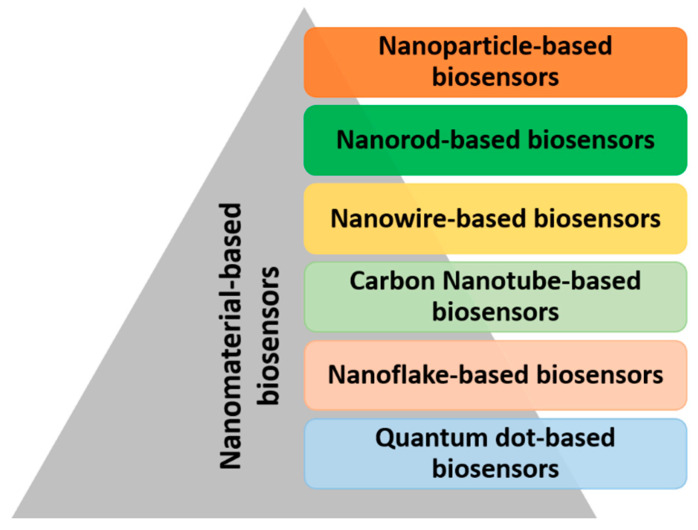
Types of nanomaterial-based biosensors (nano-biosensors).

Due to advances in nanotechnology, biosensor research has become more diverse [38]. Exploring nanomaterials based on metals and metal oxide-based nanoparticles, nanorods, nanowires, nanoflakes, nanocones, nanospherical, carbon nanotubes, quantum dots, and nanocomposites opens up the possibility to boost biosensor performance, thereby raising detection power by controlling size and shape [39,40]. Different kinds of nano-biosensors are shown in Figure 4; these work on the same principles as their macro-and micro-counterparts, but signal and data translation are performed at the nanoscale. Nano-biosensors are used for (a) physical and chemical events that are being monitored in challenging circumstances, (b) medical diagnostics and the detection of biochemicals in cellular organelles, (c) detecting nanoparticles in industrial and environmental applications, and (d) the detection of the potentially dangerous contaminants at ultra-low concentrations.

In recent years, nanoparticles (NPs) have been considered as a novel category of bioreceptors as a result of being used as transducers due to their signal-transducing abilities; various inorganic nanoparticles such as carbon nanotubes (CNTs), metal nanoparticles, and graphene have been used as transducers [41,42]. Table 2 summarizes different nanomaterials used in the development of biosensors.

#### 2.2.2. Based on Transducers

##### Calorimetric Biosensors

Thermal biosensor takes advantage of one of the most important biological responses (exothermic or endothermic), namely, the detection of a heat source absorbed or delivered. A change in temperature (DT) is proportional to the reaction enthalpy (DH) and molar number of the product (np), but inversely is proportional to mass (m) and reaction heat capacity (Cp) [59], and can be written as:

$$\mathrm{DT}=\left(\mathrm{np}\;\mathrm{DH}\right)/\mathrm{mCp}$$

Calorimetric biosensors measure the extent of a reaction or the structural dynamics of the dissolved biomolecules using heat [60]. The use of thermal sensors comprising microelectromechanical (MEMS) materials to supervise metabolic applications based on thermal monitoring has become popular lately [61].

##### Acoustic Biosensors

Acoustic biosensors work by changing the physical properties of an acoustic wave in response to a change in the amount of analyte absorbed. Because of their capacity to generate and transmit frequency-dependent acoustic waves, piezoelectric materials are frequently utilized in sensor transducers. The physical size and qualities of the piezoelectric crystal impact the ideal resonance frequency for acoustic wave propagation. Changes in the surface material mass can cause observable changes in the crystal’s inherent resonance frequency. Bulk acoustic wave (BAW) and surface acoustic wave (SAW) devices are two types of mass balance acoustic transducers.

##### Electronic Biosensors

Field-effect transistors are commonly employed in the creation of electronic biosensors (FETs). The FET is a three-terminal device that controls the current flowing through it using an electric field. These devices work between the source and drain terminals of a semiconductor, modifying the impedance that passes through the gate terminal. Furthermore, FET-based biosensors have advantages over traditional biosensing methods. However, when used in in vitro applications, these have a number of limitations. Ion-sensitive field-effect transistors (ISFETs) and metal-oxide semiconductor field-effect transistors (MOSFETs) are commonly used transistor-based sensing platforms in biological applications, depending on the technique for applying the gate voltage, design, and material of the gate and channel region. These electronic biosensors are shown to address unmet needs in other important areas, such as sustainable agriculture and environmental monitoring.

##### Electrochemical Biosensors

**Figure 5:**
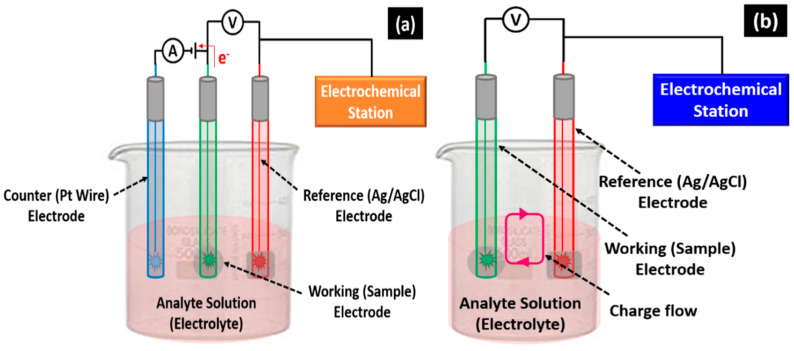

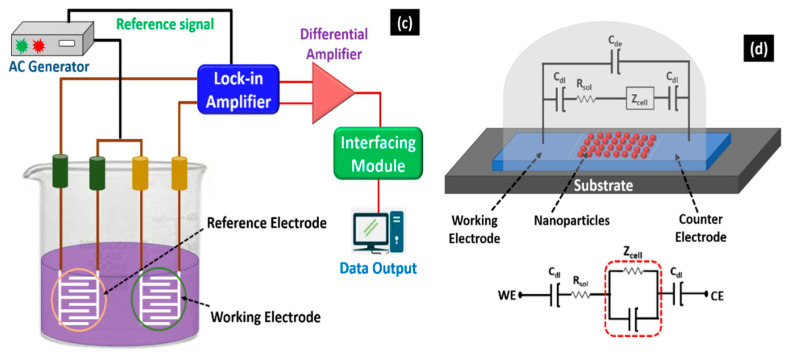
Schematic diagram of (**a**) amperometric/voltammetric, (**b**) potentiometric, and (**c**) conductometric biosensors, and (**d**) equivalent circuit of the impedimetric biosensor.

Biosensors have been fully investigated and extensively used in biological and biochemical applications [62] since these work on analyte- and transducer-based electrochemical characteristics and have good selectivity, sensitivity, and bioanalyte detection capability [63,64]. Electrochemical biosensors are classified based on their transduction mechanism: (i) potentiometric, (ii) amperometric, (iii) impedimetric, (iv) conductometric, and (v) voltammetric. Figure 5 shows a schematic representation of different types of electrochemical biosensors.

Potentiometric biosensors detect the charge accumulated at the working electrode due to analyte and bioreceptor interaction relative to the reference electrode under zero current [65,66]. When a constant voltage is given to the working electrode with respect to the reference electrode, these sensors can detect the current produced by the electrochemical oxidation or reduction in the electroactive species at the working electrode. Conductometric biosensors are used to determine how much conductance between two electrodes varies due to an electrochemical reaction, while conductometric and impedimetric biosensors are frequently used to monitor the metabolic activity in living biological systems [67]. When a small sinusoidal stimulation pulse is delivered, impedimetric biosensors can detect electrical impedance produced at the electrode/electrolyte contact. An impedance analyzer is used to quantify in/out-of-phase current response as a function of frequency when a low-amplitude AC voltage is applied to the sensor electrode [68]. Voltammetric biosensors measure the current during a regulated voltage variation to detect the analytes. These sensors have the advantages of high sensitivity readings and the simultaneous detection of several analytes [69].

Quartz crystal microbalance (QCM) is a biosensor platform that incorporates a mechanical transducer and operates on the principle of mass detection. Furthermore, it monitors changes in mass or the thickness of layers adhering to the surface of a quartz crystal [70]. Furthermore, a surface acoustic wave (SAW) biosensor produces an electromagnetic impulse signal which is sent to the device via a wired connection or wireless antenna. The electromagnetic signal is transduced into a surface acoustic wave by an interdigital transducer (IDT). These are based on horizontally polarized surface shear waves that enable the direct and label-free detection of proteins in real time. Signal response changes result mainly from mass increase and viscoelasticity changes on the device surface [71]. Table 3 shows electrochemical biosensors with principles, advantages, and disad-vantages.

#### 2.2.3. Based on Detection System

##### Optical Biosensors

An optical biosensor is a small analytical device comprising a biorecognition sensing element and an optical transducer method [72]. The fundamental goal of an optical biosensor is to provide a signal proportional to the concentration of the substance being examined. As biorecognition elements, optical biosensors can be employed for enzymes, antigens, antibodies, receptors, entire cells, nucleic acids, and tissues, among other biological materials [73]. Utilizing the interaction of the optical field and a biorecognition component, optical detection is carried out. Label-free and label-based optical biosensing can be generally divided into two general modes. Because they make it possible to detect various biological and chemical compounds directly, in real time, and without the use of labels, optical biosensors have significant benefits over traditional analytical methods. High specificity, sensitivity, compact size, and economic value are the benefits of optical biosensors. In brief, the measured signal in the label-free mode is produced directly by the interaction of the material being studied with the transducer. In contrast, label-based sensing uses a label and then uses a fluorescent, colorimetric, or luminescent approach to produce the optical signal. Surface plasmon resonance (SPR), transient wave fluorescence, and optical waveguide interferometry all employ evanescent fields close to the biosensor surface to evaluate the interaction of a biorecognition element with the analyte. Optical biosensors provide several advantages over traditional analytical techniques, including the ability to detect a variety of biological and chemical analytes directly, without the use of labels, and in real-time [74,75].

##### Mechanical Biosensors

Mechanical biosensors benefit from features that scale well when physical device size decreases, as they have a high mass resolution and the excess mass found is proportional to the total mass of the device [76]. While working in vacuum, nanoelectromechanical systems (NEMS) have achieved zeptogram-scale mass resolution and nanogram resolution in a fluid environment [77]. Mechanical biosensors are classified as: (1) affinity-based assays, which can achieve extremely selective target identification and capture using high specificity (affinity) between the target and functionalization at the device surface; (2) fingerprint tests that can identify a target based on the distinctive binding affinities to a range of sensors using a variety of less-selective functionalization layers; (3) spectrometric assays, in which target mass or optical properties are measured to facilitate the identification; or (4) separation-based assays, in which chemical interactions between the immobilized species and the flowing analytes allow the analytes to be separated spatiotemporally.

#### 2.2.4. Based on Technology

##### Miniaturized Biosensors

**Figure 6:**
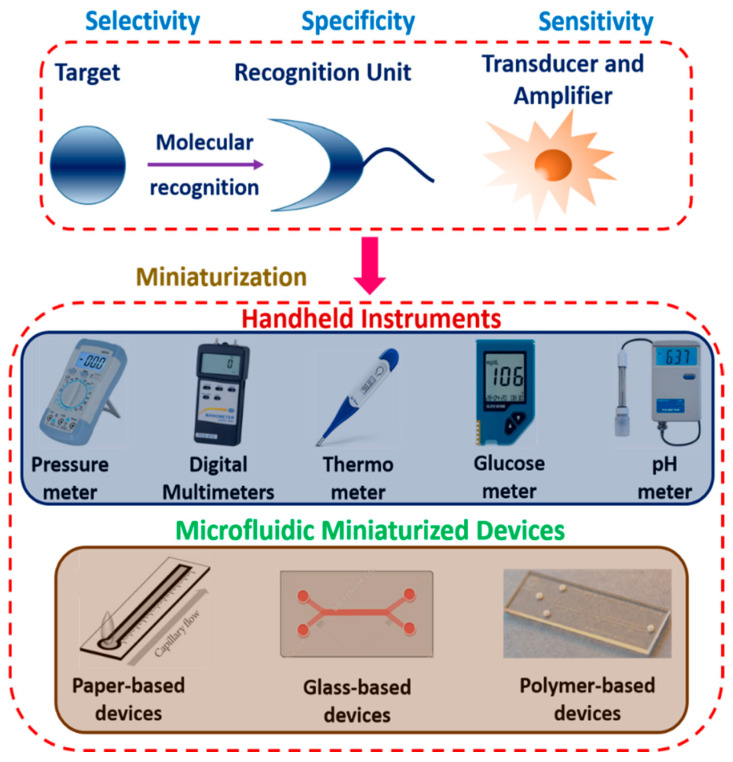
Miniaturized biosensors based on existing handheld devices and microfluidic systems for point-of-care testing (POCT).

Miniaturized biosensors for POCT have been created with advanced technology in recent years using current handheld systems and microfluidic platforms. Several basic and compact instruments such as glucometers, pressure meters, thermometers, and pH meters have proven to be helpful to determine the traditional physical characteristics [78]. New signaling pathways have been studied with small devices for low-cost, portable POC testing that does not require bulky external gear. Microfluidic systems, on the other hand, are compared to miniature biology and chemistry laboratories. These devices handle sample transmission, the mixing of solution, separation, physiological reaction, and signal output. Target recognition is an essential component in small biosensors that allows the microfluidic platform to identify various targets to evaluate sensor specificity [79]. Furthermore, miniaturized biosensors can be described based on existing handheld devices or microfluidic systems for POC testing. Figure 6 shows the schematics of miniaturized biosensors for both existing classical devices and microfluidic systems for point-of-care testing. In general, target recognition determines the specificity of the biosensor, and the transducer and amplifier determine the sensitivity of the biosensor. Miniaturized biosensors have the potential to revolutionize healthcare by measuring ions and biomolecules in real time with excellent sensitivity and precision. It has been demonstrated that these sensors are highly suited for mobile applications such as wearables, where continuous monitoring is necessary with minimum human intervention. Furthermore, three parameters such as selectivity, specificity, and sensitivity are important and play a crucial role in biosensor domains. Selectivity refers to the capability of a bioreceptor to identify a particular analyte in a real sample comprising other additives and contaminants. Specificity describes the ability of the biosensor to differentiate between target and non-targeted biological samples. Sensitivity discusses the minimum amount of an analyte that can be detected by a biosensor, defining its limit of detection (LOD).

### 2.3. Characteristics of Biosensors

Static and dynamic requirements are essential factors for highly efficient and effective biosensor devices. The functionality of biosensors can be enhanced for commercial applications. Different characteristics of biosensors include: (i) linearity, which plays a role in determining results with a better detection of regent concentration; (ii) selectivity, which is a fundamental parameter to be considered when selecting a recognition element for a biosensor; (iii) stability, which refers to the biosensing device’s susceptibility to air disturbances both inside and outside the device (the development of biological components and the degradation of biological components usually impacts stability); (iv) response time, which is the time taken for achieving >95% of the results; (v) sensitivity, which is the smallest amount of substance in a sample that can accurately be measured by an assay; and (vi) reproducibility, which is characterized by accuracy and repeatability. Table 4 discloses a list of different biosensors with working principles and applications.

## 3. Miniaturized Microfluidic-Based Biosensors: Design and Fabrication

**Figure 7:**
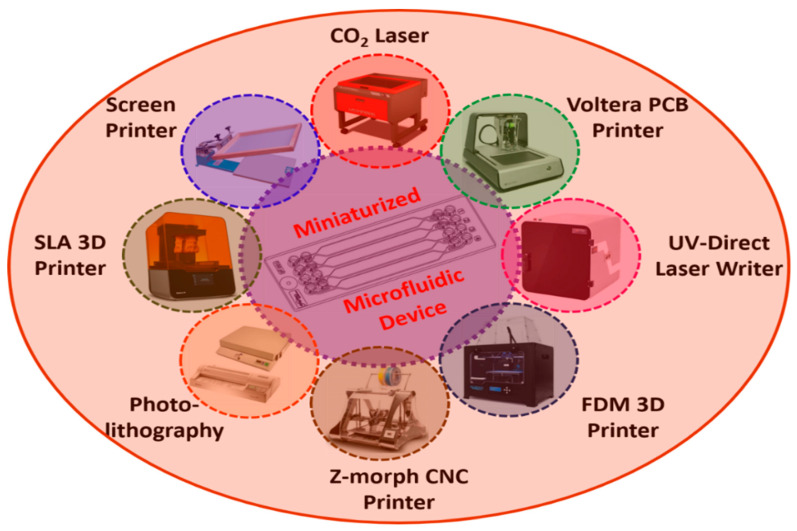
Schematic representation of various fabrication techniques used in the development of miniaturized microfluidic biosensors.

Point-of-care testing provides susceptible and non-invasive devices for detecting various biomarkers such as glucose in biological samples in a continuous, fast, low-cost, and reliable manner. Microfluidics is a form of technology that develops miniaturized biosensors using fluid mechanics. On the other hand, existing commercial devices are large, slow, expensive, and require human intervention. Despite substantial progress in developing microfluidic-based biosensors, which will continue to evolve in tandem with existing or well-established approaches, automating, integrating, and miniaturizing current conventional devices on a single platform is a challenging task. This issue has gained significant attention concerning the production of microfluidic-based small biosensors. Microfluidic devices have the advantages of mobility, operational transparency, controllability, reliability, accuracy, and stability, with a small response volume. Because of the few peripherals required to execute numerous applications, microfluidic-based biosensors fabricated using different techniques enable faster processing and greater efficiency. Figure 7 shows the schematic representation of various microfabrication techniques which are widely used in microfluidic-based biosensor development [100].

Table 5 summarizes the various microfabrication techniques used in the development of miniaturized microfluidic biosensors with different materials/substrates employed in building microfluidic devices, and their advantages and disadvantages.

### Materials

Biosensors need a critical assortment of materials to fabricate a microfluidic device. The employed materials for microfluidic channels must be sufficient and must exhibit the necessary qualities. Glass and silicon were the first materials used in microfluidic applications. Microfluidic biosensors have been developed using novel materials such as polymers, composites, and paper as time has gone on and technology has advanced. Inorganic, polymeric, and paper materials can be used to fabricate microfluidic channels. These materials ought to have a high melting point and strong thermal conductivity. Furthermore, thermal plaster or paste can be used on the microchannel in order to improve the thermal conductivity of the material. As a result, in microfluidic technology, the materials used to develop microfluidic devices are quite crucial. In general, microfluidic devices can be made from a variety of materials, such as silicone [108], glass [109], paper [110], graphene [111], polydimethylsiloxane (PDMS) [112], and polymethylmethacrylate (PMMA) [113]. Table 6 showcases several materials utilized to develop microfluidic biosensors.

**Figure 8:**
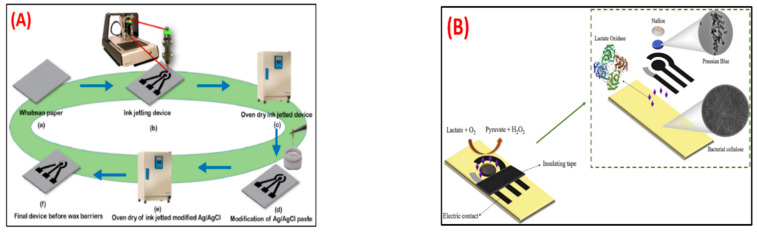
(**A**) Optimized ink-jetted paper device for electroanalytical detection of picric acid [114]. (**B**) Bacterial cellulose-based electrochemical sensing platform for miniaturized biosensors [115]. Reprinted from the above-mentioned references with the permission of copyright from the respective journals.

Jaligam et al. [114] describe a low-cost, microfluidic-based biosensor with three electrodes manufactured with an ink-jet printer on a paper substrate in which ZnO nanoparticles are drop-casted onto a working electrode (WE) to slightly modify it, as needed for the operation of cyclic voltammetry (CV) and square wave voltammetry (SWV). The effects of altering picric acid content and potential scan rates ranging from 10 to 300 mVs^−1^ were examined (Figure 8A). During the experiment, linear range was between 4 µM and 60 µM, while the detection limit was 4.04 µM, well within the safe limit of 8 µM.

Gomes et al. [115] developed a bacterial cellulose-based electrochemical sensing platform that could be used for POC sensing; this was created by utilizing the screen printing process. The substrate with bacterial cellulose displayed good resistance in terms of mechanical properties, even after measurement in an aqueous solution (Figure 8B). Lactate was measured in artificial sweat using a disposable paper-based biosensor and 50 µL of the reaction sample. The fabricated biosensor displayed an exceptional response in the amperometric method, detecting lactate in the range of 1–24 mmol L^−1^ in artificial sweat with a limit of detection of 1.31 mmol L^−1^ and a quantification limit of 4.38 mmol L^−1^.

**Figure 9:**
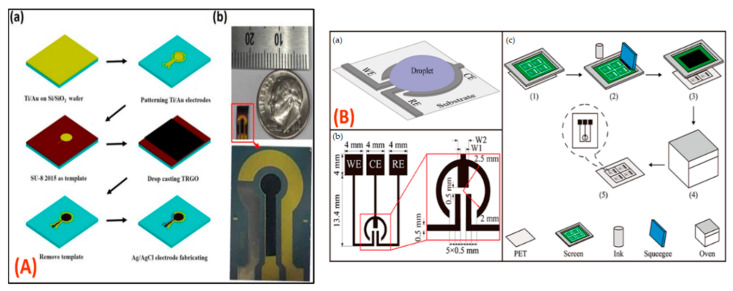
(**A**) (**a**) Schematic illustration of the fabrication of micro-patterned design with proposed heavy-metal-detection sensor with three electrodes and TRGO coating onto Au electrode; (**b**) photomicrograph of a miniaturized and completely integrated sensor [116]. (**B**) (**a**) Droplet-based electrochemical (EC) sensor design principle; (**b**) sensor component sizes and three-electrode arrangements; and (**c**) schematic illustration of the droplet-based sensor production technique using screen printing [117]. Reprinted from the above-mentioned references with permission of copyright from the respective journals.

Yong et al. [116] reported a simple and new idea for the droplet-based electrochemical sensing of several analytes associated with pharmaceutical, environmental, water, and food monitoring, making it very useful due to its cost-effectiveness and easy screen printable fabrication. The proposed biosensor has been employed in electrochemical applications in microfluidic environments (Figure 9A).

Xuan et al. [117] reported the design and fabrication of a micro-patterned, reduced graphene oxide (rGO) completely integrated with an electrochemical sensor (Figure 9B). The square wave anodic stripping voltametric (SWASV) method was used to investigate integrated electrochemical micro-ability sensors to detect the cadmium and lead ions in an acetic acid buffer solution. For both the metal ions, the micro-sensor had a detection limit of 1 g L^−1^ to 120 g L^−1^, with detection limits of 0.4 g L^−1^ and 1 g L^−1^ (S/N = 3), respectively.

**Figure 10:**
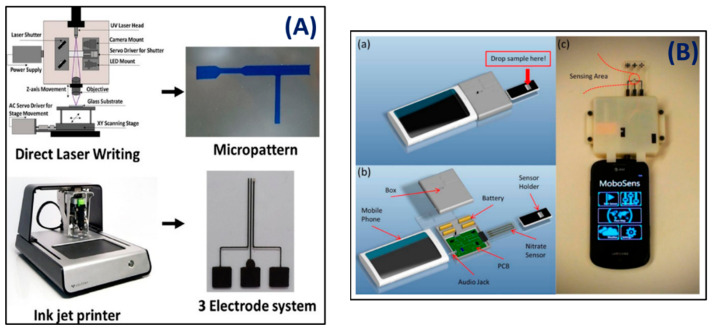
(**A**) Stepwise approach for fabricating a microfluidic device showing the use of a PCB printer to print microelectrodes and three-electrode inset [118]. (**B**) MoboSens has a concept design and a practical package: (**a**) assembly view of MoboSens, (**b**) detailed element description of MoboSens, and (**c**) photograph of the entire MoboSens system [119]. Reprinted with permission of copyright from the respective journals.

Sangam et al. [118] reported a three-electrode system fabricated with an ink-jet printer for an integrated droplet-based microfluidic device used to detect ascorbic acid electrochemically. The ink-jet printer was used to produce and integrate a microfluidic T junction device (Figure 10A). The fabricated microfluidic device was utilized to detect the ascorbic acid where accurate oxidation peaks—with a sharp peak at a potential of 0.28 V— were produced at lower flow rates (1 L/min and a combination of 2 mM concentration at 50 mV/s).

Wang et al. [119] demonstrated a smartphone-based device with an integrated easy-to-use microelectronic ionic sensor for electrochemical measurements via a smartphone audio jack (Figure 10B) for which the detection limit was 0.2 ppm; thus, the smartphone-based MoboSens platform (65 g) could measure the nitrate concentration in water in a minute. A microfabricated microfluidic sensor on the MoboSens platform detects nitrate ions using the cyclic voltammetry-based electrochemical method [120].

## 4. Applications

**Figure 11:**
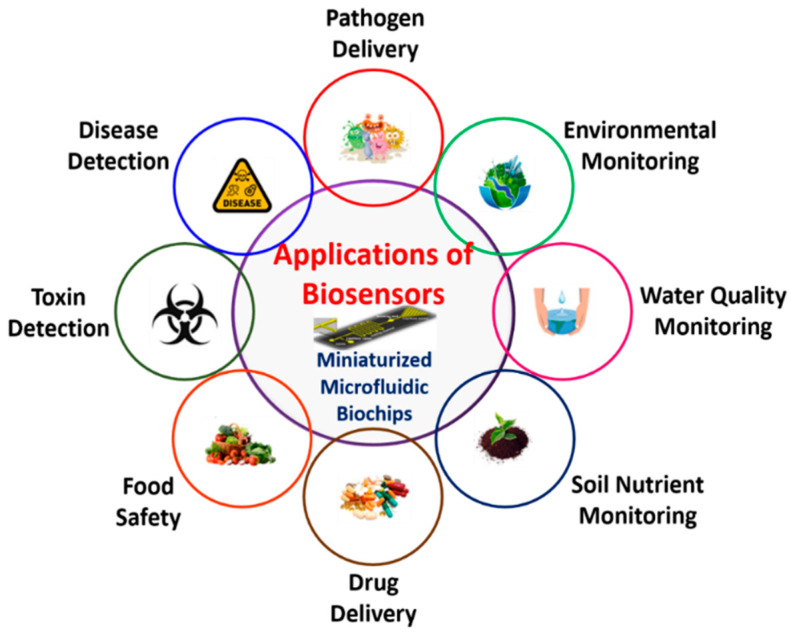
Schematic representation of various applications of biosensors.

Biosensors are utilized to increase quality of life in a variety of applications. Environmental monitoring, disease detection, food safety, defense, and drug research are applications in this category. Figure 11 shows the schematic representation of various applications of biosensors.

### 4.1. Food Processing and Environmental Monitoring

Biosensors are utilized to increase quality of life in a variety of applications. The quality, safety, and upkeep of food items as well as their processing are difficult in food processing industries, and hence the food sector would benefit from cost-effective technologies for food authentication and monitoring [121,122,123]. Potentiometric alternating biosensing devices are also used to detect the changes in pH caused by ammonia, and have been used to identify *E. coli* [124]; ingredients were mixed in a sonicator to separate the bacteria from the food [125].

Enzyme-based biosensors are also used in the dairy industry [126]; flow cells have been linked to biosensors based on screen-printed carbon electrodes, and enzymes are encapsulated by the polymer mounted onto the electrodes. Three organophosphate insecticides in milk have thus been quantified using automated flow-based biosensors [127]. Sugar substitutes are one of the most widely used food additives that are related to a number of conditions, including dental cavities, cardiovascular disease, obesity, and type II diabetes. In this case, multichannel biosensors are considered to be effective for merging lipid films via the electrochemical approaches for rapid and sensitive sweetener screening. Here, MATLAB software is used with a spatiotemporal method to evaluate the signals of glucose and sucrose in natural sugars, saccharin, and cyclamate, which represent artificial sweeteners [128].

### 4.2. Biomedical Domain

In the field of medicine, biosensor applications are fast-growing. Glucose biosensors are frequently used in clinical settings to diagnose diabetes mellitus, requiring precise blood glucose control [129]. A lateral flow assay, also known as a rapid test, is a simple device intended to detect the presence of a target substance in a liquid sample without the need for specialized and costly equipment. Around 85% of the large global market for blood glucose biosensors consists of items used at home. In the medical area, biosensors are frequently employed to diagnose infectious diseases such as urinary tract infections (UTIs), pathogen identification, and antibiotic susceptibility [130]. Human interleukin-II has been detected early by using a novel biosensor based on hafnium (IV) oxide (HfO2). For early cytokine detection, recombinant human IL-10 with a monoclonal antibody is used. The interaction between antibodies and antigens has also been studied with fluorescence patterns and electromechanical impedance spectroscopy, while fluorescence patterns have been employed to produce the bio-recognition of protein. Chen et al. [131] used HfO2 as a bio-field-effect transistor with high sensitivity [132].

### 4.3. Plant Biology

New revolutionary technologies in nucleic acid molecular imaging and sequencing have enabled advances in plant research [133]. Conventional mass spectroscopy can accurately identify cellular and subcellular localization and metabolite levels using critical data on the dynamics and location of enzymes, although transporters, substrates, and receptors still need advancement. Biosensors can access these data to quantify an active process under physiological circumstances. Strategies to envision actual processes, such as changing one metabolite into another or generating signal events, have also been attempted [133]. Tsien [134] was the first to produce protein prototype sensors for detecting caspase activity and calcium levels in living cells. These sensors were made using fluorescence resonance energy transfer (FRET) from green fluorescent protein (GFP) spectrum variants. The in vivo detection of calcium oscillations with a high temporal resolution was achieved by the use of a chameleon sensor [135].

### 4.4. Biodefense Sensing

In the case of a biological agent, biosensors can be employed for military purposes. Here, the primary goal is to promptly and correctly identify biowarfare agents (BWAs), including bacteria (vegetative and spores), poisons, and viruses. Several attempts have been made to use molecular techniques to build biosensors that can recognize the chemical markers of BWAs. Furthermore, the human papillomavirus (HPV) is a DNA virus with two strands that have been related to aggressive cervical cancer. HPV is divided into two types: HPV 16 and HPV 18 [136,137].

## 5. Limitations and Challenges in Biosensors

Biosensors have been under development for over 50 years with substantial improvements in academic and industries in the past few decades; however, only a few biosensors have reached global market success, apart from lateral flow pregnancy tests and electrochemical glucose biosensors. There are several reasons for this, such as difficulties translating academic research into commercially viable prototypes for the industry, complex regulatory issues in clinical applications, difficulty in finding qualified researchers with a background in biosensor technology, and collaboration with researchers from various science and engineering disciplines.

The existing commercial devices are large, slow, expensive, and require human intervention. Despite substantial progress in developing microfluidic-based biosensors, which will continue to evolve in tandem with the existing well-established approaches, this field has gained significant growth in the production of microfluidic-based miniaturized biosensors [138]. As a result, there are several unaddressed issues that need to be considered for the development of miniaturized commercial biosensors, including marketable biosensors that can detect specific analytes of interest that need to be identified. However, there are obvious advantages over the conventional methods for analyzing specific analytes, such as testing the biosensor performance during and after use, stability, cost, and the ease of fabricating a miniaturized biosensor.

Specificity, sensitivity, non-toxicity, small concentration detection, and cost-effectiveness are all factors in creating biosensors. Taking into account these qualities will help to solve crucial criteria and concerns about biosensor technology with significant limits. In addition, the combination of electrochemical sensors and nanomaterials has resulted in the development of novel types of biosensors. Furthermore, contact-based sensing for single analyte detection has a number of advantages, including real-time molecule measurement with high specificity. Because of differences in biomarkers between patients and related diseases, these techniques limit multiple analyte detection. However, resonant energy transfer technologies for diverse analyte detection are regularly demonstrated, which is highly rewarded in clinical diagnostics. In electrochemical sensor biofabrication, using micro- or nano-cantilevers as transducers provides a greater application potential in multiple analyte detection. Non-contact sensors that use 3D bioprinting with ink-jet or laser direct writing also perform better. The cost of these technologies, as well as their potential to be adjusted, are imperative drawbacks. However, there are significant drawbacks to these designs that make them unsuitable for widespread use. The first is the matter of background information concerning the smallest biosensors’ design and assembly.

**Figure 12:**
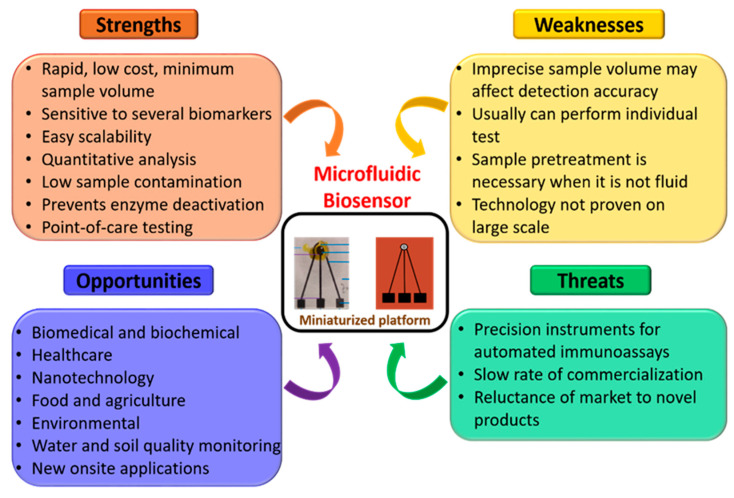
Detailed SWOT analysis of miniaturized biosensors.

Furthermore, in order to obtain a better comprehension of miniaturized biosensors, a SWOT (strength, weakness, opportunity, and threat) analysis was performed, as shown in Figure 12. It can be seen from Figure 12 that this field has outstanding strengths and unlimited opportunities in numerous domains. It can also be seen that there are areas of enhancements in respective fields that can be perfected with time and with advancement in technology. Considering the benefits of the amalgamation of this field, we firmly believe that these weaknesses can be overlooked. In addition, we trust that the collaboration of miniaturized biosensors with advanced technology, in the near future, will pave the way to achieving smart microfluidic systems for point-of-care applications.

## 6. Future Scope

The ex vivo or in vivo delivery of genetically modified proteins into cells can be used to create cell- and tissue-based biosensors. Using biophotonics or other physical principles, researchers can continually and noninvasively monitor levels of hormones, medications, or poisons. In this approach, scope could be valuable in future investigations. The market for biosensor technologies is exploding at breakneck speed. In fact, it is predicted that by 2020 it will be worth more than USD 22.5 billion. Biosensor technology is reaching new heights at the same time that demand is growing. Furthermore, biological molecules have separate structures and activities, making it difficult to combine the structure and function of nanomaterials and biomolecules to construct single-molecule multifunctional nanocomposites, nanofilms, and nanoelectrodes. Processing, testing, interface challenges, high-quality nanomaterial availability, nanomaterial tailoring, and the principles guiding the behavior of these nanoscale composites on electrode surfaces are all significant barriers for current approaches. The value chain for developing miniaturized microfluidic-based biosensor devices, including fabrication techniques and other relevant procedures, can be utilized for several POCT applications.

**Figure 13:**
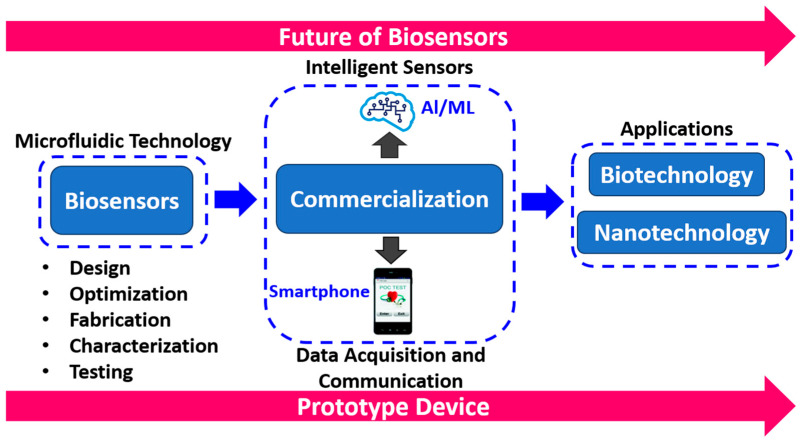
Future scope of biosensors.

Figure 13 shows the future scope of biosensors from design to commercialization. The broad scope of applications emphasizes the importance of investigating biosensors in future research. Future scope can be classified into three phases: design/development, data acquisition/communication, and application. The design/development includes the optimization, characterization and fabrication of miniaturized biosensors. Data acquisition/communication concerns graphical user interface with smartphone-enabled features. Furthermore, biosensors find a wide range of applications in the biomedical, biochemical and nanomaterial domains.

## 7. Conclusions

In recent times, there has been a drastic shift in the development of miniaturized microfluidic-based biosensors. The analysis of the interaction of biorecognition elements with biomolecular analytes, the immobilization of biomolecules on solid surfaces, the development of anti-fouling surface chemistries, fabrication, and the integration of biology with microfluidic technology, on-chip electronics, and analytical chemistry are important areas in the development of miniaturized biosensors. The rapid advances in biosensors, in terms of both research and product development, have mainly been influenced by miniaturization and microfabrication technology for better communication between life sciences and engineers/physicists. Biosensor technology is now reaching new heights, and its demand is growing. The future goals in this area should be to investigate how nanomaterials and biomolecules interact on the surface of electrodes, and how to incorporate novel features to create a new generation of biosensors with built-in advanced mechanisms to enhance sensing factors. In conclusion, producing robust, miniaturized, low-cost, automated, and easy-to-use biosensors can be helpful to mankind with an ideal mix of biosensing and biofabrication in microfluidics technology.

## Figures and Tables

**Table 1:** Evolution of the development of biosensors.

Year	Generation	Development Phases of Biosensor
1906	First	M. Cramer noticed voltage difference generating between parts of the electrolyte.
1909		Sorensen described the idea of pH and pH sensors.
1909–1922		Nelson and Griffin were the first to discover that enzyme invertase could be immobilized on charcoal aluminium hydroxide [15,16].
1922		Hughes observed a pH determination electrode [17].
1956		Clark first discovered the biosensor electrode that is capable of determining blood oxygen levels [18].
1962		Clark also demonstrated the use of an amperometric enzyme electrode for glucose sensing [19].
1967		Hicks et al. [20] enhanced Clark’s work; glucose oxidase was immobilized using an enzyme-based working electrode with an oxygen sensor.
1969		The first potentiometric enzyme electrode-based urea detection sensor was reported by Montalvo and Guilbault.
1970		Bergveld discovered ion-sensitive field-effect transistors (ISFET) [21].
1973		Lubrano and Guilbault demonstrated glucose and lactate enzyme platinum electrode to detect hydrogen peroxide (H_2_O_2_) [22].
1974		Klaus Mosbach group developed a thermistor sensor based on a heat-sensitive enzyme [23].
1975		Opitz and Lubbers developed an optical biosensor for alcohol detection [24].
1976	Second	Clemens et al. [25] integrated an electrochemical biosensor for glucose detection into an artificial bedside pancreas. A unique semi-continuous catheter-based blood glucose analyzer was also demonstrated using VIA-based technology.
1977		La Roche introduced the lactate analyzer LA 640, which was utilized to transmit an electron from dehydrogenase to an electrode [26].
1980		Peterson was the first to perform in vivo blood gas analysis to create a fiber-optic pH sensor [27].
1982		Schultz detected glucose by using the fiber-optic biosensor [28].
1983	Third	Liedberg discovered the reliance-based reactions in real time using the surface plasmon resonance (SPR) method in real time [29].
1984		For glucose detection, the first mediated amperometric biosensor was constructed using ferrocene and glucose oxidase [30].
1987		University of Cambridge created a pen-sized detector for assessing blood glucose levels.
1990		Pharmacia Biacore proposed an SPR-based biosensor [31].
1992		i-STAT developed a handheld blood biosensor [32].
2018		Girbi designed a neuron-on-chip biosensor to measure the nerve impulse conduction [33].
2021		Kulkarni et al. [34] described an Al-foil-based electrode for sensing cysteine.

**Table 2:** Nanomaterials used in the development of biosensors.

Nanoparticle	Analyte	Transducer	Linear Range	LOD	Ref
Au NPs	Aflatoxin B1	SPR Impedimetric	0.2–600 nM	0.40 nM	[43]
Au NPs	Pb^2+^	Fluorescence	40 nm–3 µm	15.9 nm	[44]
Ag NPs	H_2_O_2_ Glucose	Colorimetric	0.04–7.4 µm 1.4–3.5 µm	0.032 nm 0.29 nm	[45]
Ag/Pd NPs	Mucin 1	Electrochemi-luminescence	1.210 fg mL^−1^ −0.2110 ng mL^−1^	0.45 fg mL^−1^	[46]
Au NPs/TiO_2_	H_2_O_2_	Electrochemical	67–1525 µm	6 µm	[47]
Pt-Fe_3_O_4_@C	Sarcosine	Amperometric	0.4–62 µm	0.43 µm	[48]
Pt NFs/PANi	Urea	Cyclic Voltammetry	25 mM	10 µm	[49]
Pt@CeO_2_	Dopamine	Electrochemical	2–185 nM	0.71 nM	[50]
Cu/rGO-BP	Glucose	Electrochemical	0.3–5 mM	11 µm	[51]
Ni/Cu MOF	Glucose	FET	2 µM−25 mM	0.51 µM	[52]
NiO@Au	Lactic acid	Electrochemical	150 µM−0.6 M	11.6 µM	[53]
Co_3_O_4_	Glutamate	Electrochemical chip	12–650 µM	10 µM	[54]
MnO_2_	Salmonella	Impedimetric	3 × 10^1^–3 × 10^6^	19 CFU mL^−1^	[55]
ZnO-rGO	Dopamine	CV	0.5–1550 pM	8.75 ± 0.64 pM	[56]
ZnO NPs	Amyloid	Optoelectronic	1–15 µL	2.76 ng	[57]
TiO_2_	Asulam	Photoelectrochemical	0.04–4 ng mL^−1^	4.1 pg mL^−1^	[58]

**Table 3:** Electrochemical biosensors with principles, advantages, and disadvantages.

Electrochemical Biosensors	Principles	Advantages	Disadvantages
Potentiometric	Electric potential	Decreased analysis time, good selectivity and sensitivity, and sample treatment not required.	Temperature, pH, and immunological cross-reaction variables all have an impact on sensitivity and lifespan.
Amperometric	Oxidation/reduction	Portability due to the portable system, high selectivity, sensitivity.	Regenerative between measurements.
Impedimetric	Change in impedance	High selectivity and sensitivity, simple operation, small device.	Complex construction, expensive labelling markers.
Conductometric	Change in conductance	Low cost, fast response.	Highly buffered solution may interfere.

**Table 4:** Different types of biosensors with working principles and applications.

Types	Principles	Applications	Ref
Glucose oxidase electrode biosensor	Glucose oxidation using electrochemistry	Glucose study in biological samples.	[80]
Uric acid biosensor	Electrochemistry	The purpose of this test is to discover clinical abnormalities or diseases.	[81]
Piezoelectric biosensor	Electrochemistry	Detecting carbamate and organophosphate.	[82]
Acetylcholinesterase inhibition-based biosensor	Electrochemistry	Understanding the effects of pesticides.	[83]
HbA1c biosensor	Electrochemistry using ferroceneboronic acid	Glycated haemoglobin measurement with a robust analytical approach.	[84]
Fluorescence-tagged biosensor	Fluorescence	For a better knowledge of biological processes including the numerous molecular systems that make up a cell.	[85]
Nanoparticles-based biosensor	Electrochemical/optical/visual	Diagnostic tools are used in a variety of disciplines, including biomedicine.	[86]
Quartz–crystal biosensor	Electromagnetic	For the development of ultra-high-sensitive protein detection in liquids.	[87]
Silicon biosensor	Optical/fluorescence	Cancer therapy, bioimaging, and biosensing.	[88]
Hydrogel biosensor	Optical/visual	Biomolecular immobilization.	[89]
Microfabricated biosensor	Optical using cytochrome P450 enzyme	Pharmaceutical research and development.	[90]
Microfabricated Biosensor	Optical	To monitor biochemical oxygen demand and environmental toxicity as well as heavy metal and pesticide toxicity.	[91]
Nano-biosensors	Fiber optic	Cylindrical waveguide that guides the light within the core of the fiber used for nanomaterials and the terahertz domain meta-surface-based refractometric.	[92]
Plasmonic biosensors	Surface plasmon resonance (SPR)	Highly sensitive to the refractive index (RI) of the medium in direct contact with the metal film.	[93]
GeO_2_-doped biosensors	Refractive index (RI)	High sensitivity offers a promising approach for the detection of unknown RI analytes in chemical and biological fields in the near-infrared region.	[94]
Microchannel plasmon biosensors	Photonic crystal fiber	D-shaped photonic crystal fiber (PCF) sensor for malaria diagnosis.	[95]
MXenes-based biosensors	Fiber optic SPR sensor	A spectral SPR-based fiber optic to diagnose colorectal cancer.	[96]
Au nanowire-based biosensors	Optics	Embedded micro-drilled dual-channel approach	[97]
Au Nanowire-based biosensors	Optical Fiber Refractive Index	Concave-shaped refractive index sensor (CSRIS) exploiting localized surface plasmon resonance (LSPR).	[98]
Ag Nanowire-based biosensors	Surface plasmon resonance	Concave-shaped microfluidic channel (CSMFC).	[99]

**Table 5:** List of various microfabrication techniques used in the development of miniaturized microfluidic biosensors.

Fabrication Instruments [Ref]	Materials	Specifications	Advantages	Disadvantages
CO_2_ Laser Ablation [101]	PMMA, polyimide	IR source, λ = 10.6 µm	Precise dissection, good efficiency	Expensive instrument
Voltera Ink-jet Printer [102]	Paper, PCB, polyimide	Minimum trace width = 0.2 mm	Flexible substrates	Refilling of conductive ink
UV-Direct Laser writer (DLW) [9]	Glass, silicon wafer	GaN laser diode, λ = 405 nm	Better resolution	Expensive instrument
FDM 3D printer [103]	ABS, PLA, PCL	Filament Diameter = 1.75 mm, accuracy = 100 µm	Easily scaled to any size	Less throughput, low speed, low resolution
Z-morph 3D printer [104]	Paper, wood, PMMA	Blue laser, λ = 420 nm	Multipurpose tool with interchangeable tool heads capable of FDM 3D printing (50 µm accuracy), CNC cutting/drilling, and PCB engraving	Slow process
Photolithography [105]	Dry film photoresist (DFR)	Max width = 325 mm, maximum substrate thickness = 3 mm	Photosensitive polymers are necessary	Mask is expensive
SLA 3D printer [103]	Various liquid resins	Layer resolution = 35 microns	Higher resolution and accuracy	Requires post-processing tasks such as cleaning with IPA and ethanol
Screen printer [106]	Cloth, paper	Minimum trace width = 0.4 mm	Low cost	Less accurate
Sothlithography [107]	PDMS	Silicone elastomer	Transparent	Low thermal conductivity

**Table 6:** Different materials used for the fabrication of microfluidic biosensors.

Materials	Melting Point (°C)	Thermal Conductivity (W/mK)	Advantages	Disadvantages	Ref
Polydimethylsiloxane (PDMS)	>200 °C	2.73	Optical transparency; Low cost; Simple fabrication process; Conformal contact achievable on non-planar surfaces; Preamble to a variety of liquids and vapors; Excellent thermal stability.	Wide and shallow microchannels easily collapse during bonding; Tends to shrink to a factor of 1% upon curing.	[112]
Polymethylmethacrylate (PMMA)	150 °C	0.17–0.19	Excellent transparency; High mechanical strength and hardness; High rigidity; Good thermal stability; Low water adsorption.	Brittle; Low impact resistance; Low chemical resistance; Possibility of stress problems; Requires additional instrument to fabricate.	[113]
Graphene	>250 °C	~4000	Excellent electrical and thermal conductivity; Lightweight; Flexible; Chemically inert.	Susceptible to oxidative environment; Expensive.	[111]
Glass	1200 °C	0.76	Cheaper; Good protection power; Outstanding transparency; Great heat resistance.	Fragile; More weight.	[109]
Silicone	350 °C	0.2	Excellent thermal stability; Good flexibility; Low chemical reactivity; High efficiency.	Brittle; Expensive for a single substrate.	[108]
Paper (Cellulose)	220 °C	0.05	Very cheap; Easy to process materials; Fluid flow is automatic; Biodegradable.	Low resolution; Limited to simple designs.	[110]
